# Blood Stream Infections from MDR Bacteria

**DOI:** 10.3390/life11060575

**Published:** 2021-06-18

**Authors:** Sveva Di Franco, Aniello Alfieri, Maria Caterina Pace, Pasquale Sansone, Vincenzo Pota, Ciro Fittipaldi, Marco Fiore, Maria Beatrice Passavanti

**Affiliations:** 1 Department of Women, Child and General and Specialized Surgery, University of Campania “Luigi Vanvitelli”, 80138 Naples, Italy; svevadifranco@gmail.com (S.D.F.); anielloalfieri@gmail.com (A.A.); caterina.pace@libero.it (M.C.P.); pasquale.sansone@unicampania.it (P.S.); vincenzo.pota@unicampania.it (V.P.); marco.fiore@unicampania.it (M.F.); 2 Unit of Critical Care Hospital “Ospedale Pellegrini”, 80138 Naples, Italy; cirofittipaldi@virgilio.it

**Keywords:** bloodstream infections, intensive care unit, multidrug-resistant pathogens, septic shock

## Abstract

Background: Bloodstream infections (BSIs) constitute a growing public health concern, are among the most severe nosocomial pathologies, and are considered a worldwide cause of unfaithful outcomes, increasing treatment costs and diagnostic uncertainties. BSIs are one of the most frequent lethal conditions that are managed in intensive care units (ICUs). In the case of septic shock, immune deficiency, and delayed treatment, even with adequate antimicrobial therapy and/or source control, the outcomes are often unfavorable. Methods: this review article summarizes the epidemiological and microbiological characteristics of BSIs with a particular focus on ICU acquired BSIs (ICU-BSIs), which are usually caused by multidrug-resistant (MDR) pathogens. For this reason, their antimicrobial resistance patterns and therapeutic options have also been compiled. Results: ICU-acquired BSIs prevail in 5–7% of ICU patients. *Klebsiella pneumoniae, Escherichia coli, Acinetobacter baumannii*, and *Pseudomonas aeruginosae* are the pathogens most often responsible for MDR infections. MDR *Enterobacteriaceae* have seen their prevalence increase from 6.2% (1997–2000) to 15.8% (2013–2016) in recent years. Conclusions: Considering that prevention and treatment of sepsis is nowadays considered a global health priority by the World Health Organization, it is our obligation to invest more resources into solving or reducing the spread of these unfaithful infections. It is relevant to identify patients with risk factors that make them more susceptible to BSIs, to guarantee earlier molecular or microbiological diagnoses, and more rapidly appropriate treatment by using de-escalation strategies where possible.

## 1. Introduction

This review article summarizes the epidemiological and microbiological characteristics of bloodstream infections (BSIs) with a particular focus on intensive care unit (ICU) acquired BSIs (ICU-BSIs) caused by multidrug-resistant (MDR) pathogens, the development of resistance to antimicrobial drugs, and therapeutic strategies for empirical and targeted therapy of MDR BSIs.

BSIs are defined by positive blood culture or cultures (with an isolate of the same species grown in at least one blood culture bottle) in a patient with systemic signs of infection (i.e., a patient who has evidence of one or more of the symptoms or signs, which are fever (body temperature > 38 °C), hypothermia (body temperature < 36 °C), chills, hypotension, oliguria, or high lactate levels) [1].

BSIs constitute a growing public health concern, a life-threatening nosocomial pathology, and a worldwide primary cause of morbidity and mortality, increasing treatment costs and diagnostic uncertainties [2].

Mortality associated with BSI is 14% for BSIs developed in the community, while the rate grows to 30% in case of patients with severe comorbidities (i.e., cirrhosis, onco-hematologic diseases, or solid-organ transplants) [3,4,5].

In the case of critically ill patients, due to their high predisposition to BSIs, in the first month of hospitalization in ICUs a 7% incidence of BSIs has been reported [6].

Among this specific patient population, BSIs caused by multidrug-resistant (MDR) bacteria are a worrisome phenomenon because if they are not adequately and promptly treated, these infections are correlated with prolonged ICU stays, high costs, and poor outcomes [7].

The mortality rates are between 40% and 60%, increasing the risk of hospital death due to organ dysfunction such as sepsis or septic shock by three times. [6]

Considering that sepsis has recently been included in the global health priorities by the World Health Organization, it is our obligation to prevent this severe and unfaithful clinical evolution of BSIs [8].

## 2. Methodology

We conducted a comprehensive literature search involving several databases including PubMed, Embase, and Cochrane Library. The search terms included a combination of keywords and medical subject heading (MeSH) terms. More information on the strings used are available in Appendix A.

We followed a selection process to identify the most informative studies, analyzing titles and abstracts to retain relevant manuscripts. The full texts of the relevant manuscripts were analyzed to extract information about antibiotic resistance in bloodstream infections. Epidemiological data were obtained using Sentry Program datasets. The results of several database queries were merged and analyzed through Microsoft Excel (ver. 2019).

## 3. Epidemiology

The epidemiology of BSIs is complex, since ICU-BSIs present unique epidemiologic characteristics when compared with the BSIs that complicate both community-acquired- (CA) and hospital-acquired-(HA) infections [9].

The uniqueness of the epidemiology of BSIs, even those caused by MDR pathogens, is related to numerous factors. A mixture of different ICUs, geographical locations, antimicrobial management approaches, and the applied policies of infection control influence a BSI’s characteristics.

Worldwide, in the range of 5–7% of ICU admissions are reported to have developed a BSI there. This corresponds to a mean of 6–10 episodes per 1000 patient-days [2].

HA-BSIs in critically ill patients are community imported (i.e., documented at ICU admission) in 25% of cases, while most HA-BSI cases (75%) are acquired after admittance to the ICU [10,11].

Table 1 synthesizes the prevalence of BSIs recently reported on the SENTRY database, describing the prevalence of each pathogen in different geographical regions.

Among the pathogens causing BSIs reported in Table 1, listed in order of prevalence, we found in the first positions *K. pneumoniae* and *E. coli* with 1882 and 1747 cases of BSIs, respectively, followed by the *A. baumannii calcoaceticus species complex* and *P. aeruginosae* with 855 and 612 cases of BSIs, respectively. *Proteus mirabilis* was isolated among 315 cases, *E. cloacae species complex* in 180 cases, and *S. marcescens* in 124 cases.

According to geographical distribution in West Europe, North America, and Asia, the major prevalence is for E. coli BSIs, while in East Europe and South America the leader is *K. pneumoniae*.

Comparing the data reported in Table 1 with the data collected prior to 2008, the epidemiological trend of BSIs has dramatically changed. Between 1997 and 2004, the most common pathogen overall was *S. aureus*. Furthermore, from 2005 the prevalence of *S. aureus* resistant to methicillin (MRSA) or oxacillin (ORSA) grew until 2008 before declining from that year among community settings in all geographical regions [1].

Meanwhile, BSIs caused by extended-spectrum beta-lactamase-producing *Enterobacteriaceae* (ESBL-PE) are spreading massively worldwide.

The epidemiology of BSIs changes even according to the setting of their development.

*Escherichia coli*, *Staphylococcus aureus*, *Klebsiella pneumoniae*, and *Streptococcus pneumoniae* are the pathogens causing the largest portions of community acquired BSIs, while *Pseudomonas aeruginosae* is the cause of only 5% of community BSIs, especially in compromised patients. Patients who are immunosuppressed, who have had recent urinary tract infections, or recent pneumonia are most predisposed to *P. aeruginosae* BSIs. In this population, the prevalence of multidrug-resistant (MDR) isolates has been reported.

In the case of BSIs acquired in a hospital setting, according to the data collected from 1997 to 2016 (SENTRY network), 22% were caused by *S. aureus*, 16% by *E. coli*, 9% by K. pneumoniae, and 8% by *P. aeruginosae* [12].

The SENTRY Antimicrobial Surveillance Program, established in 1997, is one of the longest running antimicrobial surveillance networks in the world. It monitors worldwide pathogens and the changes in resistance patterns over time. The network is composed of numerous medical centers and hospital sites that participate in the program and collect data on the prevalence of different types of infections and microorganisms in their daily clinical practice. All data collected from the network are then made available and organized in the free SENTRY database.

Among the pathogens causing BSIs, the MDR species are listed in Table 2.

Between 1997 and 2016, the prevalence of MDR *Enterobacteriaceae* has increased from 6.2% to 15.8%, with a high rate of non-fermentative Gram-negative bacilli (GNB). Colistin was the only antimicrobial with a predictable 97% efficacy against *Acinetobacter Baumannii-Acinetobacter calcoaceticus complex*.

Data collected from 2013 until 2019 and available on the SENTRY database report that the most frequent MDR pathogen causing BSIs is *K. pneumoniae* with 1882 global cases (high prevalence in West Europe, East Europe and South America), followed by *Escherichia coli* with 1747 global cases (high prevalence in West Europe and North America). *A. baumannii-calcoaceticus species complex* is reported to be responsible for 855 global cases, the majority of which were in East Europe. The MDR *P. aeruginosae* caused 612 cases of BSIs, predominantly in West Europe, East Europe, and North America.

The paragraph beneath describes the MDR mechanisms and MDR species related to BSIs with a special focus on ICU acquired BSIs.

## 4. Microbiology

In healthcare settings, the emergence of MDR organisms is a major concern. The global spread and diffusion of MDR pathogens such as oxacillin-resistant *Staphylococcus aureus* (ORSA), vancomycin-resistant *Enterococcus* spp. (VRE), and MDR Gram-negative bacilli (GNB) (including extended-spectrum-β-lactamase [ESBL] producers), carbapenem-resistant *Enterobacteriaceae* (CRE), and MDR nonfermenters such as *Pseudomonas aeruginosae* and *Acinetobacter* spp. have evidenced the capability of surviving treatment with many antimicrobial agents, even the most recent ones.

This capability is due to numerous mechanisms, including alterations of cell permeability that can reduce intracellular antibiotic concentration, [13] antibiotic alteration, antimicrobial inactivation, [14] modifications to antibiotic target sites [15], and biofilm formation. We provide a quick summary of the main mechanisms of resistance in the pathogens most frequently responsible of BSIs. In Table 3, we summarize the total number of isolates by ESKAPE pathogens and the total number of BSI cases caused by these pathogens with related resistance profiles (data available on SENTRY dataset).

### 4.1. Enterococcus spp.

*Enterococci* are a family of Gram-negative bacteria that inhabit the intestines of humans and other animals. They are opportunistic pathogens capable of developing severe infections, especially *E. faecium* and *E. faecalis species* [16]. In recent years, several vancomycin-resistant strains have been identified in this family, represented mainly by E. faecium. Six types of vancomycin-resistant *Enterococcus* have been identified: Van-A, Van-B, Van-C, Van-D, Van-E, and Van-G. Van-A is the most common, showcasing the highest levels of resistance to glycopeptides [17]. The presence of these strains is usually endogenous and is easily transmitted in healthcare settings. Vancomycin-resistant enterococci had recorded an increasing incidence in North America. Data from 2002 show that more than half of the isolated enterococci strains show resistance to this. In Europe and the United Kingdom, the presence of strains resistant to vancomycin is less frequent, but in recent years the incidence of these strains has been increasing [18]. Resistance profiles of strains of *Enterococcus* spp. isolated in BSIs are shown in Table 4. 

### 4.2. Staphylococcus aureus

*S. aureus* is a Gram-positive non-motile bacterium without a notable capsule. It is normally present in the majority of adults in the skin and the mucosa of the anterior portion of the nose and pharynx. To date, the majority of isolates of this family show resistance to beta-lactam antibiotics and about 25% are methicillin-resistant [19]. However, the prevalence of methicillin-resistant *Staphylococcus aureus* (MRSA) infection may grow up to 50% in some geographical locations. In most cases, as the first-line antimicrobial, glycopeptide antibiotics (i.e., vancomycin and teicoplanin), are chosen even if the inappropriate use of these antibiotics has led some strains to become vancomycin-intermediate and vancomycin-resistant [20]. Often the vancomycin-intermediate *S. aureus* is less susceptible to teicoplanin too. VRSA, even if is less frequently encountered, is worthy of attention because of the interspecies exchange of genetic resistance genes from VRE, which results in resistances to multiple drugs [21]. Resistance profiles of strains of *S.aureus* isolated in BSIs are shown in Table 5. 

### 4.3. Klebsiella pneumoniae

*K. pneumoniae* is a Gram-negative, opportunistic pathogen. Among the members of the family Enterobacteriaceae, it is the most often found in healthcare-related infections. They are implicated in a wide range of diseases and are practically ubiquitous in nature. Infections can spread rapidly among patients hospitalized for other conditions, but the most problematic aspect is the emergence of several beta-lactamase enzymes, able to give these strains resistance to beta-lactam antimicrobials (i.e., penicillins, cephalosporins, and carbapenems). The conventional use of carbapenems to manage resolute Gram-negative infections is growing the prevalence of carbapenem-resistant *K. pneumoniae* [22]. The spread of the *K. pneumoniae* super enzyme NDM-1 (New Delhi metallo-beta-lactamase-1) has boosted the incidence of carbapenem-resistant *K. pneumoniae* isolates so that other antibiotics are rarely used [23]. The resistance profiles of strains of *K.pneumoniae* isolated in BSIs are shown in Table 6. 

### 4.4. Acinetobacter baumannii

*Acinetobacter* are Gram-negative, obligate, aerobic bacteria. They are ubiquitous and can survive up to a month on dry surfaces and are commonly found on the skin of healthcare workers, meaning that they are widely distributed in the environment and readily contaminate the hospital environment. [24] Occasionally, they can be the cause of urinary or respiratory infections in immunocompromised patients. There are many *Acinetobacter* species; all of them can cause human disease, but *Acinetobacter baumannii* is responsible for almost all infections [25].

They are resistant to numerous antibiotics because they produce beta-lactamases such as imipenem metallo-beta-lactamases and oxacillinase serine beta-lactamases which give the bacteria resistance to imipenem and colistin and, if associated, to almost all antibiotics [26]. Resistance profiles of strains of *Acinetobacter baumanii* isolated in BSIs are shown in Table 7. 

### 4.5. Pseudomonas aeruginosae

*P. aeruginosae*, as much as *Enterococci* spp., is a Gram-negative facultative anaerobe. This pathogen is usually found in the normal gut flora. If compared to others MDR pathogens it is frequently isolated in immunocompromised hospitalized patients. P. aeruginosae usually shows a propensity to develop resistance during therapy, but the isolates from ICUs show an intrinsic reduced susceptibility to several antibacterial agents above all the carbapenems. P. aeruginosae mostly develop a resistance against imipenem by the over-production of AmpC β-lactamases, the over expression of efflux pumps, the reduction of porin permeability [16], and finally the production of ESBLs (extended spectrum beta-lactamases), KPC (K. pneumoniae carbapenemase), VIM (Verona integron-encoded metallo β-lactamases), and imipenem metallo-β-lactamases. Furthermore, a combination of all the different lactamases described above can be carried by the same plasmid, leading to high rates of both carbapenem and fluoroquinolone resistance [27]. However, colistin is still effective in a high percentage of cases [26]. Resistance profiles of strains of *P.aeruginosae* isolated in BSIs are shown in Table 8. 

### 4.6. Enterobacter spp.

*Enterobacter* spp. are facultative, anaerobic, motile Gram-negative bacteria. There are normally commensals present in the gut of man, but they can cause infections in immunosuppressed or hospitalized subjects. Many strains exhibit numerous resistance mechanisms, such as extended-spectrum beta-lactamase, metallo-beta-lactamase and carbapenemases, which confer resistance to almost all antibiotics except tigecycline and colistin [28]. The resistance profiles of strains of *Enterobater* spp. isolated in BSIs are shown in Table 9.

## 5. Risk Factors

Due to the increased interest during recent years on the topic of ICU-BSIs, several risk factors have been defined that may be used to promptly recognize and treat susceptible patients. The APACHE II score (Acute Physiologic Assessment and Chronic Health Evaluation II score), prolonged in-hospital stay, need for mechanical ventilation, renal replacement therapy, immunosuppression, compromised liver functionality, surgery, invasive procedures, and acquired infections have been identified as the main risk factors for BSIs [29].

Among acquired infections, it has recently been demonstrated that even the COVID-19 disease is strictly related to poor outcomes in hospitalized patients, predisposing patient to infection by MDR in-hospital pathogens with low survival rates even though there is currently no therapy for COVID-19 infection whose efficacy has been proven [30,31].

**Figure 1:**
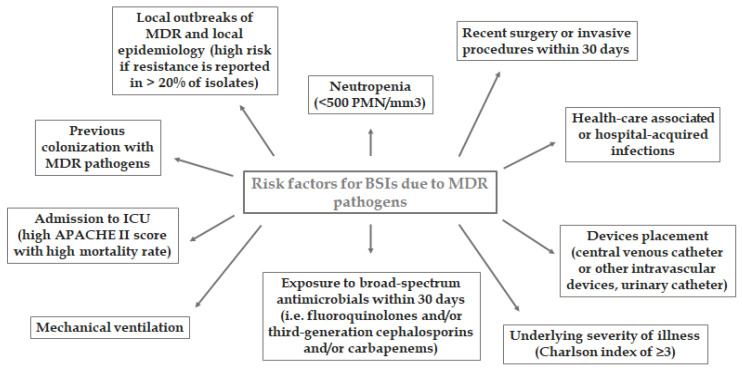
Risk factors for BSIs due to MDR pathogens.

The risk factors are listed in Figure 1.

According to the EUROBACT-1 international study that enrolled 1156 ICU-acquired BSIs, 21% of infections originated from venous catheters (catheter-related BSIs (CR-BSIs)), 21% were form nosocomial pneumonia (ventilator associated pneumonia (VAP)) (which is a frequent complication when mechanical ventilation is required), 24% of episodes were due to intra-abdominal infections, and another 24% had no definite source [10].

The diagnosis of CR-BSIs is provided by the identification of the same microbiological pathogen from both peripheral blood cultures and from the culture of the venous catheter tips. CR-BSIs are among the most frequently encountered BSIs in ICU patients, along with BSIs related to VAP and/or to abdominal infections, including the development from the urinary tract [29].

## 6. Early Microbiological Diagnosis in BSI

Even if culture methods represent the best choice for detecting an infection, the methodology based on molecular assays is achieving remarkable results in terms of specificity and execution times. In the context of sepsis, in fact, timing is crucial and antibiotic therapy should be changed abruptly based on laboratory results. Molecular assays offer rapid results on blood samples without prior incubation. These new techniques are able to determine pathogens and related resistances but, unfortunately, still show a medium sensitivity for pathogens and have a limited number of antibiotic resistances [32];

Besides, a prompt initiation of empirical antimicrobial therapy may be the only chance for a septic patient, but may also significantly reduce the sensitivity of blood cultures drawn, even shortly after treatment initiation [33].

The choice of antimicrobial agent for empirical therapy must take into account several factors such as: the type of pathogen suspected of being involved, any suspicion of resistance or the onset of fungal infection [34,35].

Leukopenia and immunosuppression are other factors to consider because they increase the risk of MDR and fungal infections [36].

Recently, new magnetic resonance-based tests have been introduced that show good sensitivity and short execution times (T2Bacteria Panel, T2Biosystems) [37].

Other very promising, but in development, methods to obtain quickly an etiological accurate diagnosis are next-generation sequencing (NGS) and application of machine-learning [38,39,40].

These techniques may effectively improve treatment optimization in the ICU, reducing the percentage of empirically treated infections [36], anticipating the timing of de-escalation treatment, and improving critically ills patients’ outcomes [41].

In this scenario, a thrifty use of recently approved drugs active against MDR organisms is fundamental. The objective of treatment should be to promptly administrate an effective treatment, not improving the selection of antimicrobial resistance using the most recent and high spectrum drugs indiscriminately [42]. Therefore, the prevalence of carbapenemases in each clinical environment should now be taken into account when prompting empirical therapies. The availability of novel beta-lactams/beta-lactamases inhibitor (BL-BLI) combinations, active against MDR Gram-negative bacteria expressing different determinants of resistance, is already changing the approach to management of septic patients [43].

## 7. Rationale of Treatment

### 7.1. Single-Drug or Combination Therapy for Bloodstream Infection in ICU Patients

Nowadays, in the case of a patient with a diagnosis of a blood stream infection the primary object when planning a first line empirical treatment regimen is to combine multiple antimicrobial molecules to maximize the likelihood of efficacy against the hypothesized pathogen due to the high rates of antimicrobial resistance. Usually, the associations of antimicrobials include a beta-lactam plus an aminoglycoside or a beta-lactam plus a fluoroquinolone. However, once the pathogen responsible of the infection has been identified and the profile of resistance detected, the choice of whether to continue with an association therapeutic regime or to switch to a targeted regime with a single antimicrobial is arbitrary, and little and conflicting evidence has been made available to date. According to experimental models, antimicrobial combination can prevent or postpone the selection of resistant species. The synergistic action of several antibiotics has to be exploited in case of BSIs by *P. aeruginosae* and/or other non-fermenting Gram-negative pathogens [44]. The lack of clinical reports confirming the data collected from in vitro models leaves unsettled the utility of combination therapy to prevent antimicrobial resistance development. Furthermore, numerous studies and meta-analyses were not able to demonstrate that the association of beta-lactam and aminoglycosides or fluoroquinolones in comparison to beta-lactam monotherapy can reduce fatality rates in patients, including those with sepsis or neutropenia [45]. Moreover, in a regimen that uses a beta-lactam antibiotic, the introduction of an aminoglycoside has frequently increased the rate of acute renal failure in the acute phase of infection [45,46].

Even on a pathogen-specific analysis, in the case of BSI due to methicillin-susceptible *S. aureus* (except in those with implanted devices) or *Enterobacterales*, including AmpC-hyperproducers and ESBL-PE, there is poor data to demonstrate that a double antimicrobial regimen favorably impacts patient outcomes [1].

In the case of carbapenem-resistant *A. baumanni*, a polymyxin-based combination may perform better than polymyxin alone only when a high-dose colistin regimen is administered.

Concerning BSIs caused by *P. aeruginosae*, strong doubts as to the advantages of combination therapy persist, because no rise in survival rates has been detected yet [1].

Recently, two systematic reviews evaluated combination therapy based on Ceftolozane-Tazobactam or Ceftazidime-Avibactam compared to monotherapies for the Treatment of Severe BSIs [47].

In conclusion, combination therapy is still an indicated approach for patients with septic shock, but should not be prescribed as routine treatment. Conditions other than severe infections, including sepsis without circulatory failure, may not benefit from antimicrobial combination but may suffer from cumulative side effects [26].

### 7.2. De-Escalation Strategy

In the contest of antimicrobial stewardship strategies (AMS), antimicrobial de-escalation (ADE) is a strategy that aims to reduce the spectrum of the chosen antibiotic, narrowing its spectrum but not reducing treatment efficacy, and to decrease the emergence of antimicrobial resistance—even reducing the number of antimicrobials involved in treatment [48]. The ADE should be started 2–3 days after diagnosis of an infection; with the availability of microbiological specimens, the re-evaluation of antimicrobial regimens can be performed. Considering that in all BSIs, the pathogen or the pathogens are always known, these infections are perfect candidates for re-evaluation. According to ADE strategy, the source and the pathogen responsible of the BSI are isolated, and it is strictly recommended, even in immunocompromised patients [49], to stop broad spectrum combination therapy and to re-evaluate the treatment regimen. For example, in case of a diagnosis of a Gram-negative BSI, anti-MRSA or antifungal agents should be suspended because their administration has been proven to be ineffective.

In the case of ADE, regarding the antibiotic chosen empirically as a first line molecule, the management will be more complex due to multiple factors.

The antibiotics’ spectrum of action is variable according to the region of the world, and the ranking depends on the priorities that are considered [50].

The period of in-hospital stay and the comorbidities of the patient are factors that surely will influence the development of antimicrobial resistance. The employment of ADE usually lengthens the duration of antimicrobial therapy [51]. Since multiple recent studies on different sources of infection have recommended a shorter duration of antimicrobial therapy as a target of treatment because longer exposure to antimicrobials predisposes one to the development of MDR pathogens [52].

Sometimes the switching from beta-lactam to oral fluoroquinolones may be useful at ward dismissal to reduce in-hospital patient stay, but this strategy may not be so useful in the ICU due to the high rate of resistance that has emerged from using those therapeutic regimens.

Carbapenems are the most used antimicrobials in ICU therapeutics regimens, however the incidence of resistance has increased, especially in the case of long course treatment and, unfortunately, most pathogens that have become endemic in ICUs have developed multiple resistance mechanisms to this class of antimicrobials, therefore MDR pathogens have been found even after only 1–3 days of in-ICU therapy [53]. According to what was said before about the early development of resistance, this renders ADE useless.

In some cases, another factor that influences antimicrobial management is patients’ antimicrobial flora, which may conditionate the emergence of resistance and the response to treatment [54].

In the case of polymicrobial infections (i.e., intra-abdominal infections), it is important to be cautious because not all pathogens are evidenced by blood cultures, and drugs not continued according to ADE may have been required.

Using in silico pharmacokinetic–pharmacodynamic (PK/PD) modeling, it has been shown that the conventional dosing strategy of using a narrow spectrum beta-lactam may have higher risks of not attaining the target compared to broad spectrum regimens [55].

Furthermore, it must be considered that some narrower spectrum alternatives are sometimes more effective than broad-spectrum regimens (i.e., oxacillin or cephazolin are superior to piperacillin/tazobactam in *S. aureus* BSIs) [56].

It is strictly recommended that one consider all the points described above before deciding whether narrowing the first line antimicrobial is the adequate decision to take in the case of BSIs in critically ill patients. The ADE is spreading among clinicians as a main part of the global AMS re-evaluation plan, with the objective of the optimization of the treatment in patients with a severe infection. The ADE consent to adapt antimicrobial treatment of BSIs every time the laboratory data elaboration provides new information on the profile of the pathogens that are the cause of infections.

## 8. Conclusions

BSIs are frequent conditions that need to be diagnosed and treated in ICUs and in-hospital patients. BSIs are associated with impaired outcomes, especially in the case of sepsis/septic shock, immune deficiency, and delayed adequate antimicrobial therapy. Among severely ill patients, the prevalence of MDR pathogens is higher when compared to other classes of hospitalized patients. The increase in healthcare related BSIs due to MDR pathogens may stress the need for innovative diagnostic tools that can improve the fast and accurate identification of resistance markers.

While waiting for such data, the choice regarding the best empirical regimen to be promptly adopted depends on many clinical parameters, including the patient’s individual risk factors, individual predisposition to MDR infections, and the geographical pathogen distribution.

Combination therapy might be a solution capable of improving survival rates in the most severely ill patients, even if further data need to be collected and analyzed to validate this theory. Once culture results become available, the source of a BSI is identified and well controlled, antimicrobial de-escalation can be performed, and treatment duration can be shortened as much as possible. Longer treatments may be proposed only in specific clinical scenarios such as BSI due to *S. aureus*.

## Figures and Tables

**Table 1:** Number of reported cases of BSIs according to pathogens and geographical distribution.

Pathogens Causing BSIs	Reported Cases of BSIs for Country (n = Number of Cases)					
	World n. BSIs/n. Tot (66,729/319,581)	Asia n. BSIs/n. Tot (6914/29,359)	West Europe n. BSIs/n. Tot (20,897/77,554)	East Europe n. BSIs/n. Tot (6689/29,313)	South America n. BSIs/n. Tot (5188/19,462)	North America n. BSIs/n. Tot (27,041/163,893)
***K. pneumoniae***	1882	150	551	561	335	285
***Escherichia coli***	1747	266	612	285	164	420
***Acinetobacter baumannii-calcoaceticus species complex***	855	98	188	345	155	69
***Pseudomonas aeruginosae***	612	41	172	175	75	149
***Proteus mirabilis***	351	13	142	50	14	132
***E. cloacae species complex***	180	22	22	18	48	70
***S. marcescens***	124	2	33	34	32	23
***E. cloacae***	114	12	44	23	14	21
***Morganella morganii***	87	3	23	10	6	45
***K. oxytoca***	59	1	21	8	8	21
***P. stuartii***	54		12	9	4	29
***Klebsiella aerogenes***	41	5	15	5	3	13
***C. freundii species complex***	25	3	8	1	1	12
***Citrobacter freundii***	14		7			7
***Hafnia alvei***	14		9	1		4
***A. lwoffii***	7			2	2	3
***A. pittii***	7	1	2	2		2
***Providencia rettgeri***	5				1	4
***Unspeciated acinetobacter***	5	1		2		2
***A. berezinae***	4			3	1	
***A. nosocomialis***	3	1	1			1
***A. ursingii***	3					3
***Enterobacter asburiae***	3	1		1		1
***A. johnsonii***	2	1	1			
***C. koseri***	2		1			1
***P. vulgaris group***	2		2			
***Acinetobacter baumannii***	1				1	
***A. radioresistens***	1					1
***E. hormaechei***	1		1			
***K. variicola***	1	1				
***Pluralibacter gergoviae***	1		1			
***P. vulgaris***	1					1
***Raoultella ornithinolytica***	1					1
***Serratia liquefaciens***	1				1	
***S. rubidaea***	1					1
***Providencia (unspeciated)***	1				1	
***Raoultella (unspeciated)***	1					1
***Salmonella (unspeciated)***	1	1				
***Serratia (unspeciated)***	1				1	

**Table 2:** MDR bacteria causing BSIs from SENTRY database.

MDR Bacteria Causing BSIs Form SENTRY Database						
Pathogen	World	Asia	West Europe	East Europe	North America	South America
***K. pneumoniae***	1882	150	551	561	285	335
***Escherichia coli***	1747	266	612	285	420	164
***A. baumannii-calcoaceticus species complex***	855	98	188	345	69	155
***Pseudomonas aeruginosae***	612	41	172	175	149	75
***Proteus mirabilis***	351	13	142	50	132	14
***E. cloacae species complex***	180	22	22	18	70	48
***Serratia marcescens***	124	2	33	34	23	32
***E. cloacae***	114	12	44	23	21	14
***Morganella morganii***	87	3	23	10	45	6
***K. oxytoca***	59	1	21	8	21	8
***Providencia stuartii***	54		12	9	29	4
***Klebsiella aerogenes***	41	5	15	5	13	3
***C. freundii species complex***	25	3	8	1	12	1
***Citrobacter freundii***	14		7		7	
***Hafnia alvei***	14		9	1	4	
***A. lwoffii***	7			2	3	2
***A. pittii***	7	1	2	2	2	
***Providencia rettgeri***	5				4	1
***unspeciated Acinetobacter***	5	1		2	2	
***A. berezinae***	4			3		1
***A. nosocomialis***	3	1	1		1	
***A. ursingii***	3				3	
***Enterobacter asburiae***	3	1		1	1	
***A. johnsonii***	2	1	1			
***C. koseri***	2		1		1	
***P. vulgaris group***	2		2			
***Acinetobacter baumannii***	1					1
***A. radioresistens***	1				1	
***E. hormaechei***	1		1			
***K. variicola***	1	1				
***Pluralibacter gergoviae***	1		1			
***P. vulgaris***	1				1	
***Raoultella ornithinolytica***	1				1	
***Serratia liquefaciens***	1					1
***S. rubidaea***	1				1	
***Providencia (unspeciated)***	1					1
***Raoultella (unspeciated)***	1				1	
***Salmonella (unspeciated)***	1	1				
***Serratia (unspeciated)***	1					1

**Table 3:** Summary of the number of isolates by ESKAPE pathogens and the total number of BSI cases due to these pathogens with related resistance profiles (data available on SENTRY dataset).

Pathogen	n. Cases of Infections (n = Number)	n. BSIs Reported (n = Number)	Frequent Resistance Profiles in BSIs
***Enterococcus* spp.**	15,022	5154	Van-A
***S. aureus***	69,918	13,608	MRSA (less frequent VRSA)
***K. pneumoniae***	26,701	6901	NMD-1
***Acinetobacter* spp.**	7151	1372	IMP and OXA
***P. aeruginosae***	28,096	3264	ESBLs, KPC, VIM and IMP
***Enterobacter* spp.**	11,597	2241	ESBLs

Abbreviations—Van-A: vancomycin and teicoplanin resistance pattern; MRSA: methicillin-resistant *S. aureus*; VRSA: vancomycin resistant *S. aureus*; NMD-1: New Delhi metallo-beta-lactamase; IMP: imipenem metallo-beta-lactamases; OXA: oxacillinase serine beta-lactamases; ESBLs: extended spectrum beta-lactamases; KPC: K. pneumoniae carbapenemase; VIM: Verona integron-encoded metallo β-lactamases.

**Table 4:** *Enterococcus* spp. isolates from bloodstream infections’ resistance profiles.

Agent	MIC50	MIC90	Range	Count	CLSI ^a^				EUCAST ^a^			
					%S	%I	%R	Count	%S	%I	%R	Count
Amoxicillin-clavulanic acid	≤1	>8	≤1 to >8	1451								
Piperacillin-tazobactam	8	>16	≤2 to >16	3042					64.9	0.3	34.8	3040
Imipenem	1	>8	≤0.12 to >8	1458								
Meropenem	8	>8	0.25 to >8	1458								
Cefepime	>16	>16	≤0.5 to >16	1458								
Ceftaroline	4	>8	≤0.25 to >8	5045								
Ceftriaxone	>8	>8	≤0.06 to >8	1458								
Trimethoprim-sulfamethoxazole	≤0.5	>4	≤0.5 to >4	1458								
Teicoplanin	≤2	>16	≤2 to >16	5145	85.3	2.1	12.7	5145	84.7		15.3	5145
Vancomycin	1	>16	≤0.5 to >16	5145	83.1	0.6	16.3	5145	83.1		16.9	5145
Tigecycline	≤0.06	0.12	≤0.06 to >0.5	5142					99.6		0.4	4971
Clindamycin	>2	>2	≤0.25 to >2	2246								
Daptomycin	1	2	≤0.25 to >8	5144								
Erythromycin	>16	>16	≤0.12 to >16	2245	8.2	27.5	64.2	2245				
Linezolid	1	2	≤0.25 to >8	5145	99.6	0.2	0.2	5145	99.8		0.2	5145
Ampicillin	1	>8	≤0.5 to >8	5150	66.0		34.0	5150	65.7	0.4	34.0	5150
Penicillin	4	>8	≤0.06 to >8	1448	61.8		38.2	1448				
Ciprofloxacin	>4	>4	≤0.03 to >4	1457	36.8	8.6	54.6	1457	47.4		52.6 ^b^	1457
Levofloxacin	4	>4	≤0.5 to >4	5151	48.4	2.0	49.6	5151	50.4		49.6 ^b^	5151
Moxifloxacin	2	>4	≤0.25 to >4	4262								
Doxycycline	8	>8	≤0.06 to >8	1353	46.0	34.5	19.5	1353				
Minocycline	8	>8	≤1 to >8	3691	48.1	19.9	32.1	3691				
Tetracycline	>8	>8	≤1 to >8	5141	32.9	1.0	66.1	5141				

^a^ Criteria as published by CLSI (2021) and EUCAST (2021), ^b^ Uncomplicated UTI only. Organisms included: *Enterococcus avium* (20), *E. casseliflavus* (44), *E. cecorum* (1), *E. durans* (11), *E. faecalis* (3002), *E. faecium* (1980), *E. gallinarum* (68), *E. hirae* (13), *E. mundtii* (2), and *E. raffinosus* (13). Report generated from MVP (https://sentry-mvp.jmilabs.com, assessed on 4 June 2021), a product of JMI Laboratories, on 5 June 2021 11:06:30 GMT/UTC. The data and information are available in the SENTRY Antimicrobial Surveillance Program dataset.

**Table 5:** *Staphylococcus aureus* isolates from bloodstream infections’ resistance profiles.

Agent	MIC50	MIC90	Range	Count	CLSI ^a^				EUCAST ^a^			
					%S	%I	%R	Count	%S	%I	%R	Count
Amoxicillin-clavulanic acid	≤1	>8	≤1 to >8	4055	65.7		34.3	4055				
Piperacillin-tazobactam	1	>16	≤0.5 to >16	8387	66.0		34.0	8387	66.0		34.0	8387
Imipenem	≤0.12	>8	≤0.12 to >8	4021	65.8		34.2	4021				
Meropenem	0.12	>8	≤0.06 to >8	4023	65.8		34.2	4023				
Cefepime	4	>16	≤0.5 to >16	4026	65.8		34.2	4026				
Ceftaroline	0.25	1	≤0.06 to 8	13,099	95.8	4.2	0.1 ^b^	13,099	95.8	4.1	0.1 ^c^	13,099
									95.8		4.2 ^d^	13,099
Ceftriaxone	4	>8	≤0.25 to >8	13,608	66.3		33.7	13,608				
Trimethoprim-sulfamethoxazole	≤0.5	≤0.5	≤0.5 to >4	13,608	98.3		1.7	13,608	98.3	0.2	1.4	13,608
Teicoplanin	≤2	≤2	≤2 to 16	13,606	>99.9	<0.1	0.0	13,606	99.6		0.4	13,606
Vancomycin	1	1	≤0.12 to 2	13,607	100.0	0.0	0.0	13,607	100.0		0.0	13,607
Tigecycline	0.06	0.12	≤0.015 to 1	13,602	>99.9			13,602	>99.9		<0.1	13,602
Clindamycin	≤0.25	>2	≤0.25 to >2	13,608	87.6	0.1	12.3	13,608	87.4	0.2	12.4	13,608
Daptomycin	0.25	0.5	≤0.12 to 4	13,607	99.9			13,607	99.9		0.1	13,607
Azithromycin	0.5	>4	≤0.03 to >4	5034	59.2	1.2	39.6	5034	58.4	0.8	40.8	5034
Erythromycin	0.25	>8	≤0.12 to >8	13,603	59.1	4.4	36.5	13,603	59.5	1.6	38.9	13,603
Linezolid	1	2	≤0.12 to 4	13,607	100.0		0.0	13,607	100.0		0.0	13,607
Oxacillin	0.5	>2	≤0.25 to >2	13,608	66.3		33.7	13,608	66.3		33.7	13,608
Penicillin	>2	>2	≤0.06 to >2	8377	17.5		82.5 ^e^	8377	17.5		82.5	8377
Ciprofloxacin	0.5	>4	0.06 to >4	4021	68.4	1.8	29.7	4021				
Levofloxacin	0.25	>4	≤0.12 to >4	13,604	71.4	0.5	28.1	13,604				
Moxifloxacin	≤0.12	4	≤0.12 to >4	11,069	72.0	5.6	22.4	11,069	71.6		28.4	11,069
Doxycycline	≤0.06	0.25	≤0.06 to >8	13,102	98.1	1.6	0.3	13,102	95.2	1.8	3.1	13,102
Minocycline	≤0.06	0.12	≤0.06 to >8	9581	98.9	0.6	0.5	9581	97.7		2.3	9581
Tetracycline	≤0.5	1	≤0.5 to >8	13,602	92.6	0.8	6.6	13,602	91.3	0.6	8.1	13,602

^a^ Criteria as published by CLSI (2021) and EUCAST (2021). ^b^ Intermediate is interpreted as susceptible-dose dependent. ^c^ Using other than pneumonia breakpoints. ^d^ Using pneumonia breakpoints. ^e^ Oxacillin non-susceptible is reported as resistant. Organisms included: *Staphylococcus aureus* (13,608). The data and information are available in the SENTRY Antimicrobial Surveillance Program dataset.

**Table 6:** *Klebsiella pneumoniae* isolates from bloodstream infections’ resistance profiles.

Agent	MIC50	MIC90	Range	Count	CLSI ^a^				EUCAST ^a^			
					%S	%I	%R	Count	%S	%I	%R	Count
Amikacin	1	8	≤0.25 to >32	6897	93.5	3.0	3.5	6897	91.3		8.7 ^b^	6897
Gentamicin	≤1	>8	≤1 to >8	6899	81.6	0.9	17.5	6899	81.0		19.0 ^b^	6899
Tobramycin	0.5	>8	≤0.12 to >8	6897	74.1	4.8	21.1	6897	72.5		27.5 ^b^	6897
Amoxicillin-clavulanic acid	4	>8	≤1 to >8	4662	67.6	8.6	12.1	4662				
Ampicillin-sulbactam	8	>32	≤0.5 to >32	6901	56.4	7.0	36.7	6901	56.4		43.6 ^c^	6901
Cefoperazone-sulbactam	≤0.25	>32	≤0.25 to >32	5723	78.1		21.9 ^d^	5723				
Ceftazidime-avibactam	0.12	1	≤0.015 to >32	1074	98.5		1.5	1074	98.5		1.5	1074
Ceftolozane-tazobactam	0.25	>16	≤0.12 to >16	1073	84.5	1.4	14.1	1073	84.5		15.5	1073
Piperacillin-tazobactam	4	>64	≤0.5 to >64	6891	78.1	5.1	16.8	6891	71.9		28.1	6891
Doripenem	≤0.12	2	≤0.12 to >4	5827	89.5	0.8	9.7	5827	89.5	0.8	9.7	5827
Ertapenem	0.015	>2	≤0.008 to >2	2888	85.2	1.0	13.8	2888	85.2		14.8	2888
Imipenem	≤0.12	2	≤0.12 to >8	6899	89.4	1.0	9.6	6899	90.4	1.2	8.4	6899
Meropenem	≤0.06	2	≤0.06 to >8	6899	89.4	0.8	9.8	6899	90.2		9.8 ^e^	6899
									90.2	1.7	8.0 ^f^	6899
Cefepime	≤0.5	>16	≤0.5 to >16	6899	70.9	2.5	26.6 ^g^	6899	69.9	2.0	28.1	6899
Cefoperazone	0.5	>32	≤0.25 to >32	1450	68.1	0.9	31.0 ^d^	1450				
Cefoxitin	4	>16	1 to >16	883	77.5	4.6	17.9	883				
Ceftaroline	0.12	>16	≤0.03 to >16	6738	65.0	2.0	33.0	6738	65.0		35.0	6738
Ceftazidime	0.25	>16	≤0.12 to >16	6901	70.3	2.3	27.4	6901	68.4	1.9	29.7	6901
Ceftriaxone	≤0.06	>8	≤0.06 to >8	6899	68.8	0.6	30.6	6899	68.8		31.2 ^e^	6899
									68.8	0.6	30.6 ^f^	6899
Cefuroxime	4	>64	≤0.5 to >64	2458	53.5	8.5	37.9 ^h^	2458	58.8		41.2 ^j^	2458
					58.8	3.3	37.9 ^i^	2458				
Trimethoprim-sulfamethoxazole	≤0.5	>4	≤0.5 to >4	6893	67.0		33.0	6893	67.0	1.1	31.9	6893
Tigecycline	0.5	1	≤0.06 to 8	6893	98.0	1.7	0.2 ^k^	6893				
Colistin	≤0.5	≤0.5	≤0.5 to >8	6848					95.8		4.2	6848
Polymyxin B	1	1	≤0.25 to >8	1757								
Aztreonam	≤0.12	>16	≤0.12 to >16	6899	70.5	0.8	28.7	6899	69.2	1.3	29.5	6899
Ciprofloxacin	≤0.03	>4	≤0.03 to >4	6884	65.2	4.1	30.7	6884	65.2	4.1	30.7	6884
Levofloxacin	≤0.12	>4	≤0.12 to >4	6884	70.9	5.0	24.1	6884	70.9	5.0	24.1	6884
Moxifloxacin	≤0.25	>4	≤0.25 to >4	5465					60.3		39.7	5465
Doxycycline	2	>8	0.12 to >8	6372	69.5	6.6	23.9	6372				
Minocycline	2	>8	≤0.06 to >8	6521	78.8	7.7	13.4	6521				
Tetracycline	2	>8	≤0.5 to >8	6526	68.6	3.8	27.6	6526				

^a^ Criteria as published by CLSI (2021) and EUCAST (2021). ^b^ For infections originating from the urinary tract. For systemic infections, aminoglycosides must be used in combination with other active therapy. ^c^ These breakpoints for oral administration are relevant for uncomplicated urinary tract infections only. ^d^ The cefoperazone breakpoints were applied following US FDA criteria. ^e^ Using meningitis breakpoints. ^f^ Using non-meningitis breakpoints. ^g^ Intermediate is interpreted as susceptible-dose dependent. ^h^ Using oral breakpoints. ^i^ Using parenteral breakpoints. ^j^ Using oral, uncomplicated urinary tract infection only breakpoints. ^k^ US FDA breakpoints were applied. Organisms included: *Klebsiella pneumoniae* (6901). Report generated using MVP (https://sentry-mvp.jmilabs.com, assessed on 4 June 2021), a product of JMI Laboratories, on 5 June 2021 11:09:47 GMT/UTC. The data and information are available in the SENTRY Antimicrobial Surveillance Program dataset.

**Table 7:** Acinetobacter isolates from bloodstream infections’ resistance profiles.

Agent	MIC50	MIC90	Range	Count	CLSI ^a^				EUCAST ^a^			
					%S	%I	%R	Count	%S	%I	%R	Count
Amikacin	>32	>32	≤0.25 to >32	1370	46.4	2.6	50.9	1370	44.7		55.3 ^b^	1370
Gentamicin	>8	>8	≤1 to >8	1370	44.7	3.9	51.4	1370	44.7		55.3 ^b^	1370
Tobramycin	2	>8	≤0.12 to >8	1370	56.2	1.2	42.6	1370	56.2		43.8 ^b^	1370
Amoxicillin-clavulanic acid	>8	>8	≤1 to >8	666								
Ampicillin-sulbactam	32	>32	≤0.5 to >32	1372	39.2	8.1	52.7	1372				
Cefoperazone-sulbactam	16	>32	≤1 to >32	1184								
Piperacillin-tazobactam	>64	>64	≤0.5 to >64	1359	33.3	3.4	63.3	1359				
Doripenem	>4	>4	≤0.12 to >4	1201	39.6	0.6	59.8	1201				
Imipenem	>8	>8	≤0.12 to >8	1370	41.9	0.6	57.5	1370	41.9	0.6	57.5	1370
Meropenem	>8	>8	≤0.06 to >8	1370	40.4	1.1	58.5	1370	40.4		59.6 ^c^	1370
									40.4	2.0	57.6 ^d^	1370
Cefepime	>16	>16	≤0.5 to >16	1370	35.5	4.4	60.1	1370				
Cefoperazone	>32	>32	8 to >32	378								
Ceftazidime	>16	>16	≤0.25 to >16	1372	34.4	3.9	61.7	1372				
Ceftriaxone	>8	>8	0.25 to >8	1026	17.1	0.0	0.0	1026				
Trimethoprim-sulfamethoxazole	4	>4	≤0.5 to >4	1371	48.1		51.9	1371	48.1	2.5	49.5	1371
Tigecycline	1	4	≤0.12 to >8	1370								
Colistin	≤0.5	2	≤0.5 to >8	1368					92.1		7.9	1368
Polymyxin B	1	2	≤0.25 to >8	443								
Aztreonam	>16	>16	0.25 to >16	1372								
Ciprofloxacin	>4	>4	≤0.03 to >4	1370	37.3	0.4	62.3	1370				
Levofloxacin	>4	>4	≤0.12 to >4	1372	38.6	3.7	57.7	1372	37.6	0.4	62.0	1372
Moxifloxacin	>4	>4	≤0.25 to >4	1122								
Doxycycline	1	>8	≤0.06 to >8	1124	65.4	1.3	33.3	1124				
Minocycline	0.5	>8	≤0.06 to >8	1348	75.8	6.7	17.5	1348				
Tetracycline	>8	>8	≤0.5 to >8	1165	39.5	9.8	50.7	1165				

^a^ Criteria as published by CLSI (2021) and EUCAST (2021). ^b^ For infections originating from the urinary tract. For systemic infections, aminoglycosides must be used in combination with other active therapy. ^c^ Using meningitis breakpoints. ^d^ Using non-meningitis breakpoints. Organisms included: *Acinetobacter baumannii* (1), *A. baumannii-calcoaceticus species complex* (1134), *A. beijerinckii* (1), *A. berezinae* (12), *A. courvalinii* (2), *A. guillouiae* (5), *A. haemolyticus* (2), *A. johnsonii* (12), *A. junii* (16), *A. lwoffii* (44), *A. nosocomialis* (6), *A. pittii* (18), *A. radioresistens* (24), *A. schindleri* (4), *A. soli* (3), *A. towneri* (1), *A. ursingii* (60), *A. variabilis* (6), and *unspeciated Acinetobacter* (21). The data and information are available in the SENTRY Antimicrobial Surveillance Program dataset.

**Table 8:** *Pseudomonas aeruginosa* isolates from bloodstream infections’ resistance profiles.

Agent	MIC50	MIC90	Range	Count	CLSI ^a^				EUCAST ^a^			
					%S	%I	%R	Count	%S	%I	%R	Count
Amikacin	4	16	≤0.25 to >32	3264	92.6	1.5	5.9	3264	92.6		7.4 ^b^	3264
Gentamicin	2	>8	≤1 to >8	3264	86.0	3.5	10.5	3264				
Tobramycin	0.5	>8	≤0.12 to >8	3264	88.8	0.6	10.6	3264	88.4		11.6 ^b^	3264
Ampicillin-sulbactam	>32	>32	4 to >32	3264								
Cefoperazone-sulbactam	4	>32	≤1 to >32	2743								
Ceftazidime-avibactam	2	8	0.25 to >32	428	93.9		6.1	428	93.9		6.1	428
Ceftolozane-tazobactam	0.5	2	≤0.12 to >16	435	93.8	0.9	5.3	435	93.8		6.2	435
Piperacillin-tazobactam	4	>64	≤0.5 to >64	3259	79.3	10.0	10.7	3259				
Doripenem	0.5	>4	≤0.12 to >4	2827	80.3	6.0	13.7	2827				
Imipenem	1	>8	≤0.12 to >8	3264	75.6	3.8	20.6	3264				
Meropenem	0.5	>8	≤0.06 to >8	3254	78.4	5.3	16.3	3254	78.4		21.6 ^c^	3254
									78.4	10.1	11.4 ^d^	3254
Cefepime	2	16	≤0.5 to >16	3256	83.5	8.9	7.6	3256				
Cefoperazone	8	>32	0.25 to >32	768								
Ceftazidime	2	>16	≤0.25 to >16	3257	81.1	4.4	14.5	3257				
Trimethoprim-sulfamethoxazole	4	>4	≤0.5 to >4	3257								
Colistin	1	2	≤0.5 to >8	3264					99.3		0.7	3264
Polymyxin B	2	2	≤0.25 to 4	937								
Aztreonam	8	>16	0.25 to >16	3257	70.3	11.8	17.8	3257				
Ciprofloxacin	0.12	>4	≤0.03 to >4	3259	76.5	3.5	20.0	3259				
Levofloxacin	0.5	>4	≤0.12 to >4	3253	69.9	7.3	22.8	3253				
Moxifloxacin	1	>4	≤0.25 to >4	2626								
Minocycline	>8	>8	0.25 to >8	3164								

^a^ Criteria as published by CLSI (2021) and EUCAST (2021). ^b^ For infections originating from the urinary tract. For systemic infections, aminoglycosides must be used in combination with other active therapy. ^c^ Using meningitis breakpoints. ^d^ Using non-meningitis breakpoints. Organisms included: *Pseudomonas aeruginosa* (3264). Report generated using MVP (https://sentry-mvp.jmilabs.com, assessed on 4 June 2021), a product of JMI Laboratories, on 5 June 2021 11:13:28 GMT/UTC. The data and information are available in the SENTRY Antimicrobial Surveillance Program dataset.

**Table 9:** *Enterobacter* spp. isolates from bloodstream infections’ resistance profiles.

Agent	MIC50	MIC90	Range	Count	CLSI ^a^				EUCAST ^a^			
					%S	%I	%R	Count	%S	%I	%R	Count
Amikacin	1	2	≤0.25 to >32	2239	98.6	0.3	1.1	2239	97.9		2.1 ^b^	2239
Gentamicin	≤1	≤1	≤1 to >8	2241	92.5	0.8	6.7	2241	91.7		8.3 ^b^	2241
Tobramycin	0.5	4	≤0.12 to >8	2238	90.9	1.9	7.2	2238	89.6		10.4 ^b^	2238
Amoxicillin-clavulanic acid	>8	>8	≤1 to >8	1451	3.3	1.4	57.5	1451				
Ampicillin-sulbactam	32	>32	0.5 to >32	2233	17.2	20.1	62.7	2233	17.2		82.8 ^c^	2233
Cefoperazone-sulbactam	0.5	16	≤0.25 to >32	1853	82.6		17.4 ^d^	1853				
Ceftazidime-avibactam	0.25	0.5	0.03 to 32	337	99.7		0.3	337	99.7		0.3	337
Ceftolozane-tazobactam	0.25	8	≤0.12 to >16	337	84.3	3.0	12.8	337	84.3		15.7	337
Piperacillin-tazobactam	2	64	≤0.5 to >64	2237	81.1	9.8	9.1	2237	77.2		22.8	2237
Doripenem	≤0.12	≤0.12	≤0.12 to >4	1903	98.1	0.5	1.4	1903	98.1	0.5	1.4	1903
Ertapenem	0.06	0.5	≤0.008 to >2	883	90.8	4.5	4.6	883	90.8		9.2	883
Imipenem	0.25	1	≤0.12 to >8	2240	97.8	0.8	1.4	2240	98.6	0.4	1.0	2240
Meropenem	≤0.06	0.12	≤0.06 to >8	2240	98.3	0.3	1.4	2240	98.6		1.4 ^e^	2240
									98.6	0.7	0.7 ^f^	2240
Cefepime	≤0.5	4	≤0.5 to >16	2238	88.8	4.3	6.9 ^g^	2238	83.1	8.5	8.4	2238
Cefoperazone	0.5	>32	≤0.25 to >32	471	76.2	1.9	21.9 ^d^	471				
Cefoxitin	>16	>16	2 to >16	286	3.1	1.7	95.1	286				
Ceftaroline	0.25	>16	≤0.03 to >16	2200	66.4	3.7	29.9	2200	66.4		33.6	2200
Ceftazidime	0.5	>16	≤0.12 to >16	2241	73.6	1.1	25.3	2241	69.8	3.7	26.4	2241
Ceftriaxone	0.25	>8	≤0.06 to >8	2219	68.4	1.5	30.1	2219	68.4		31.6 ^e^	2219
									68.4	1.5	30.1 ^f^	2219
Cefuroxime	16	>64	0.5 to >64	646	12.7	44.4	42.9 ^h^	646				
					43.5	13.6	42.9 ^i^	646				
Trimethoprim-sulfamethoxazole	≤0.5	>4	≤0.5 to >4	2237	84.4		15.6	2237	84.4	0.2	15.4	2237
Tigecycline	0.5	1	≤0.06 to >8	2240	98.6	1.4	0.1 ^j^	2240				
Colistin	≤0.5	>8	≤0.5 to >8	2166					78.8		21.2	2166
Polymyxin B	1	>8	≤0.25 to >8	539								
Aztreonam	≤0.12	>16	≤0.12 to >16	2235	74.0	1.7	24.3	2235	71.5	2.5	26.0	2235
Ciprofloxacin	≤0.03	1	≤0.03 to >4	2235	85.7	3.1	11.2	2235	85.7	3.1	11.2	2235
Levofloxacin	≤0.12	1	≤0.12 to >4	2237	89.4	2.5	8.2	2237	89.4	2.5	8.2	2237
Moxifloxacin	≤0.25	2	≤0.25 to >4	1732					79.3		20.7	1732
Doxycycline	2	8	≤0.06 to >8	2039	87.7	5.2	7.0	2039				
Minocycline	2	8	0.25 to >8	2057	88.9	4.8	6.3	2057				
Tetracycline	2	>8	≤0.5 to >8	2069	85.1	3.2	11.7	2069				

^a^ Criteria as published by CLSI (2021) and EUCAST (2021). ^b^ For infections originating from the urinary tract. For systemic infections, aminoglycosides must be used in combination with other active therapy. ^c^ These breakpoints for oral administration are relevant for uncomplicated urinary tract infections only. ^d^ The cefoperazone breakpoints were applied following US FDA criteria. ^e^ Using meningitis breakpoints. ^f^ Using non-meningitis breakpoints. ^g^ Intermediate is interpreted as susceptible-dose dependent. ^h^ Using oral breakpoints. ^i^ Using parenteral breakpoints. ^j^ US FDA breakpoints were applied. Organisms included: Enterobacter asburiae (21), E. cancerogenus (2), E. cloacae (1182), E. cloacae species complex (1027), E. hormaechei (3), E. intermedius (1), and E. kobei (5). Report generated using MVP (https://sentry-mvp.jmilabs.com accessed on 4 June 2021), a product of JMI Laboratories, on 5 June 2021 11:14:36 GMT/UTC. The data and information are available in the SENTRY Antimicrobial Surveillance Program dataset.

## Appendix A

**Table A1:** Search Strings.

Database	Search String	No. of Results
PUBMED	(“anti infective agents”[Pharmacological Action] OR “anti infective agents”[MeSH Terms] OR (“anti infective”[All Fields] AND “agents”[All Fields]) OR “anti infective agents”[All Fields] OR “antimicrobial”[All Fields] OR “antimicrobials”[All Fields] OR “antimicrobially”[All Fields]) AND (“resist”[All Fields] OR “resistance”[All Fields] OR “resistances”[All Fields] OR “resistant”[All Fields] OR “resistants”[All Fields] OR “resisted”[All Fields] OR “resistence”[All Fields] OR “resistences”[All Fields] OR “resistent”[All Fields] OR “resistibility”[All Fields] OR “resisting”[All Fields] OR “resistive”[All Fields] OR “resistively”[All Fields] OR “resistivities”[All Fields] OR “resistivity”[All Fields] OR “resists”[All Fields]) AND (“sepsis”[MeSH Terms] OR “sepsis”[All Fields] OR (“bloodstream”[All Fields] AND “infections”[All Fields]) OR “bloodstream infections”[All Fields])	13,466
EMBASE	(‘bloodstream infection’/exp OR ‘blood infection’ OR ‘blood infections’ OR ‘blood stream infection’ OR ‘blood stream infections’ OR ‘blood-borne infections’ OR ‘bloodstream infection’ OR ‘bloodstream infections’) AND (‘antibiotic resistance’/exp OR ‘antibacterial drug resistance’ OR ‘antibacterial resistance’ OR ‘antibiotic non-susceptibility’ OR ‘antibiotic nonsusceptibility’ OR ‘antibiotic resistance’ OR ‘antimicrobial drug resistance’ OR ‘antimicrobial resistance’ ODatabase Search String N. of result PUBMED (“anti infective agents”[Pharmacological Action] OR “anti infective agents”[MeSH Terms] OR (“anti infective”[All Fields] AND “agents”[All Fields]) OR “anti infective agents”[All Fields] OR “antimicrobial”[All Fields] OR “antimicrobials”[All Fields] OR “antimicrobially”[All Fields]) AND (“resist”[All Fields] OR “resistance”[All Fields] OR “resistances”[All Fields] OR “resistant”[All Fields] OR “resistants”[All Fields] OR “resisted”[All Fields] OR “resistence”[All Fields] OR “resistences”[All Fields] OR “resistent”[All Fields] OR “resistibility”[All Fields] OR “resisting”[All Fields] OR “resistive”[All Fields] OR “resistively”[All Fields] OR “resistivities”[All Fields] OR “resistivity”[All Fields] OR “resists”[All Fields]) AND (“sepsis”[MeSH Terms] OR “sepsis”[All Fields] OR (“bloodstream”[All Fields] AND “infections”[All Fields]) OR “bloodstream infections”[All Fields]) 13,466 EMBASE (‘bloodstream infection’/exp OR ‘blood infection’ OR ‘blood infections’ OR ‘blood stream infection’ OR ‘blood stream infections’ OR ‘blood-borne infections’ OR ‘bloodstream infection’ OR ‘bloodstream infections’) AND (‘antibiotic resistance’/exp OR ‘antibacterial drug resistance’ OR ‘antibacterial resistance’ OR ‘antibiotic non-susceptibility’ OR ‘antibiotic nonsusceptibility’ OR ‘antibiotic resistance’ OR ‘antimicrobial drug resistance’ OR ‘antimicrobial resistance’ OR ‘bacterial drug resistance’ OR ‘bacterial resistance’ OR ‘bacterium resistance’ OR ‘drug resistance, bacterial’ OR ‘drug resistance, microbial’ OR ‘microbial drug resistance’ OR ‘resistance, antibiotic’) 4558 Cochrane bloodstream infections and antimicrobial resistance 71R ‘bacterial drug resistance’ OR ‘bacterial resistance’ OR ‘bacterium resistance’ OR ‘drug resistance, bacterial’ OR ‘drug resistance, microbial’ OR ‘microbial drug resistance’ OR ‘resistance, antibiotic’)	4558
Cochrane	bloodstream infections and antimicrobial resistance	71
